# Equivalence of conventional and fast late gadolinium enhancement (LGE) techniques for quantitative evaluation of fibrosis in ischemic and non-ischemic cardiac disease - Save the Time!

**DOI:** 10.1186/1532-429X-18-S1-Q64

**Published:** 2016-01-27

**Authors:** Fabian Muehlberg, Kristin Arnhold, Stephanie Funk, Marcel Prothmann, Andre Rudolph, Florian von Knobelsdorff-Brenkenhoff, Jeanette Schulz-Menger

**Affiliations:** Working Group on Cardiovascular MRI, Charité University Medicine and HELIOS Clinics, Berlin, Germany

## Background

Segmented single-slice/single-breath-hold 2D phase-sensitive inversion recovery (2D-PSIR) sequences are the gold standard for evaluation of myocardial fibrosis. Aim of this study was to assess the accuracy of novel free-breathing or single-breath-hold LGE sequences to detect and quantify myocardial fibrosis in patients with different entities.

## Methods

**Figure 1:**
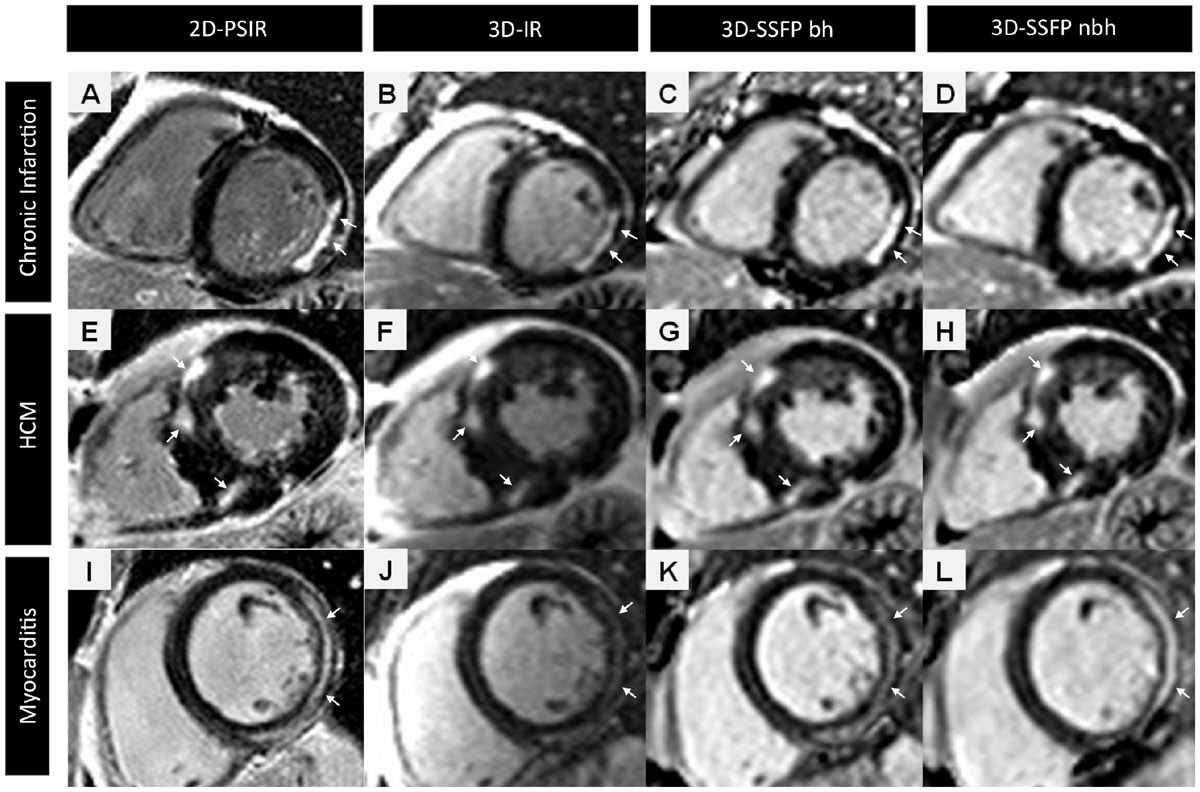
**LGE images of three patients with chronic ischemis infarction (A-D), HCM (E-H) and acute myocarditis (I-L)**. Arrows indicate typical LGE localization. Horizontal rows display corresponding slices of LGE in the same patient using conventional segmented 2d-PSIR (A;E;I), 3D-IR (B;F;J), 3D-SSFP bh (C; G;K) and 3D-SSFP nbh 9D;H;L).

Patients with myocardial infarction (n = 45), myocarditis (n = 25) or hypertrophic cardiomyopathy (HCM) (n = 15) were prospectively enrolled. After administration of gadolinium contrast agent, LGE images were acquired ECG-gated in short axis slices (slice thickness 7 mm, no gap) using 4 different LGE sequences: (1) conventional segmented 2D phase-sensitive inversion recovery in single-slice/single-breath-hold technique (2D-PSIR; gold standard; TR 744 ms, TE 5,17 ms, voxel size 1.4 × 1.4 × 7.0 mm), (2) single-breath-hold 3D-IR sequence (3D-IR bh; TR 924 ms, TE 1.06 ms, voxel size 1.9 × 1.9 × 7.0 mm), (3) single breath-hold 3D-SSFP sequence (3D-SSFP; TE 700 ms, TE 1.05 ms, voxel size 1.9 × 1.9 × 7.0 mm) and (4) non-breath-hold technique (3D-IR nbh). (Figure 1) For all techniques, inversion time was individually adjusted to null the remote myocardium. Myocardial fibrosis was quantitatively assessed using a semi-automated threshold method; positive LGE was defined as signal intensity 6 standard deviations (SD) above signal intensity of remote myocardium for myocardial infarction and 3 SD for myocarditis / HCM. Detection rates were determined as number of matching myocardial AHA segments with detected LGE in gold standard and each fast technique.

## Results

Overall detection rates of fibrosis - compared to the gold standard - were not significantly lower for any of the fast LGE sequences: 3D-IR (83.06 ± 20.0%), 3D-SSFP bh (88.25 ± 18.5%), and 3D-SSFP nbh (86.48 ± 14.7%).

There was no significant difference in size of myocardial fibrosis between the segmented 2D-PSIR, the 3D-IR and 3D-SSFP sequence (Figure 2), independent of the underlying etiology. Correlation of infarct size in each fast sequence was significant towards gold standard, i.e. for myocardial infarction (3D-IR: r^2^ = 0.801; p = 0.01/3D-SSFP bh: r^2^ = 0.851; p = 0.01/3D-SSFP nbh: r^2^ = 0.834; p = 0.01), acute myocarditis (3D-IR: r^2^ = 0.788; p = 0.01/3D-SSFP bh: r^2^ = 0.949; p = 0.01/3D-SSFP nbh: r^2^ = 0.944; p = 0.01) or HCM (3D-IR: r^2^ = 0.904; p = 0.01/3D-SSFP bh: r^2^ = 0.905; p = 0.01/3D-SSFP nbh: r^2^ = 0.938; p = 0.01).

**Figure:**
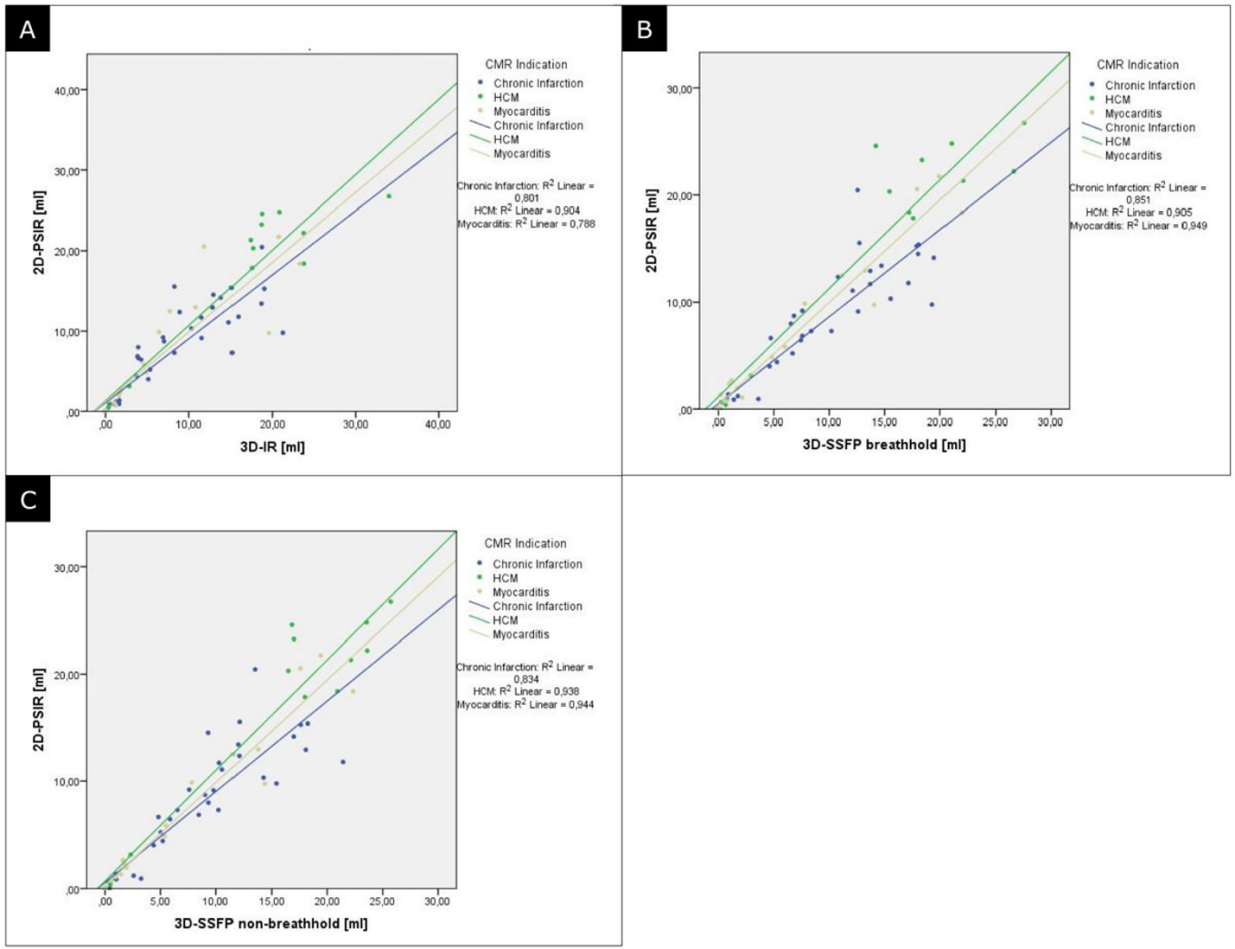
Figure 2

Acquisition times were significantly shorter for 3D-IR (23.2 s ± 8.2 s) and 3D-SSFP (21.8 s ± 7.2 s) as compared to 2D-PSIR (375.5 s ± 86.3 s).

## Conclusions

Fast 3D-SSFP, 3D-IR and conventional segmented 2D-PSIR sequences are equivalent techniques for the assessment of myocardial fibrosis, independent of an ischemic or non-ischemic etiology. Due to the minimized acquisition time they shorten scan protocols by up to 6 minutes.

